# Epidural Co-Administration of Dexmedetomidine and Levobupivacaine Improves the Gastrointestinal Motility Function after Colonic Resection in Comparison to Co-Administration of Morphine and Levobupivacaine

**DOI:** 10.1371/journal.pone.0146215

**Published:** 2016-01-11

**Authors:** Xian-Zhang Zeng, Zhi-Fang Lu, Xiang-Qi Lv, Yue-Ping Guo, Xiao-Guang Cui

**Affiliations:** Department of Anesthesiology, Second Hospital of Harbin Medical University, 246 Xuefu Road, Harbin, 150001, Heilongjiang, China; The Chinese University of Hong Kong, HONG KONG

## Abstract

Gastrointestinal motility may be impaired after intestinal surgery. Epidural morphine is effective in controlling postoperative pain, but can further reduce gastrointestinal motility. Here, we aimed to investigate the effects of epidural dexmedetomidine on gastrointestinal motility in patients undergoing colonic resection. Seventy-four patients undergoing colonic resection were enrolled in this clinical trial and allocated randomly to treatment with dexmedetomidine (D group) or morphine (M group). The D group received a loading dose epidural administration of 3 ml dexmedetomidine (0.5 μg kg^-1^) and then a continuous epidural administration of 80 μg dexmedetomidine in 150 ml levobupivacaine (0.125%) at 3 ml h^-1^ for two days. The M group received a loading dose epidural administration of 3 ml morphine (0.03 mg kg^-1^) and then a continuous epidural administration of 4.5 mg morphine in 150 ml levobupivacaine at 3 ml h^-1^ for two days. Verbal rating score (VRS), postoperative analgesic requirements, side effects related to analgesia, the time to postoperative first flatus (FFL) and first feces (FFE) were recorded. VRS and postoperative analgesic requirements were not significantly different between treatment groups. In contrast, the time to FFL and time to FFE were significant longer in M group in comparison to D group (P < 0.05). Moreover, patients in M group had a significantly higher incidence of nausea, vomiting, and pruritus (P < 0.05). No patients showed neurologic deficits in either group. In comparison to morphine, epidural dexmedetomidine is safe and beneficial for the recovery of gastrointestinal motility after colonic resection when used as an adjunct with levobupivacaine for postoperative pain control.

**Trial Registration:**

Chinese Clinical Trial Registry ChiCTR-TRC-14004644

## Introduction

Gastrointestinal motility is commonly impaired after intestinal surgery,[1] which causes an increased incidence of postoperative ileus.[2] Co-administration of epidural morphine and local anaesthetic is a common and effective method of postoperative pain control. However, postoperative epidural morphine is associated with further reductions in gastrointestinal motility. Thus, improved epidural anaesthetic management for postoperative pain control that does not impair gastrointestinal motility would be beneficial.

Dexmedetomidine is a centrally selective α2-adrenoceptor agonist that can be given as either a sedative or an analgesic without significant respiratory depression.[3] In recent years, dexmedetomidine has been administered for either spinal or epidural anesthesia[4–7] in combination with local anaesthetics. In these studies, dexmedetomidine potentiated local anesthesia and had few side effects. In our previous study, epidural dexmedetomidine not only potentiated the effects of local anesthetics, but also shortened the time of first flatus of patients after nephrectomy.[8] Although statistical significance was not observed in this pilot study, the trend was compelling and warranted further study. Furthermore, this trend is supported by animal studies.[9] For example, investigators have found that dexmedetomidine showed beneficial effects on the recovery of intestinal motility in rats.[9] However, some disagreement in the literature exists regarding the effects of dexmedetomidine.[10, 11] Thus, the benefits of epidural dexmedetomidine remain largely unknown, although some studies suggest that dexmedetomidine is preferential to morphine in terms of gastrointestinal motility.

From these studies, we hypothesized that dexmedetomidine influences gastrointestinal motility, and speculated that differences in the dose and route of administration of dexmedetomidine may largely explain the contradictory reports present within the literature. The objective of the present study was to investigate the effects of low dose epidural dexmedetomidine on gastrointestinal motility after colonic resection in comparison to co-administration of morphine and levobupivacaine. The primary efficacy endpoint of the study was the time to postoperative first flatus, a variable reflecting gastrointestinal motility.

## Materials and Methods

### Study subjects

This randomised, double blind, prospective, controlled study was approved by the Ethics Committee of Harbin Medical University, China (approval number: HMUIRB20140004) and registered with the Chinese Clinical Trial Registry at www.chictr.org on 14 May 2014 (registration number: ChiCTR-TRC-14004644). With written consent, 74 American Society of Anesthesiology Physical Status I/II patients undergoing elective colonic resection in the Second Hospital of Harbin Medical University between May 2014 and December 2014 were enrolled. To eliminate any possible effects of surgical technique, all procedures were undertaken by a single surgeon. The surgeries were performed through a midline laparotomy; all anastomoses were performed using mechanical staplers. Patients received standardized care during the perioperative period, and were allowed to ingest small amounts of water orally during the first 24h post-operative. After 24h post-operative, patients were allowed ingest semi-solid food. Progression to a normal diet was not allowed until first flatus occurred. No subjects had a history of any of the following conditions: neurologic or psychiatric illness, allergy to local anaesthetic agents, diabetes, gastrointestinal motility disorder, prior abdominal surgery, renal or hepatic insufficiency, bleeding or coagulation abnormalities, or anticoagulant therapy.

### Treatment groups

Using a computer-generated random number table, the patients were randomly assigned to Dexmedetomidine group (D group) or Morphine group (M group) and received different postoperative analgesia plans following surgical completion. Patients in the D group had the following treatment regimen: a loading dose epidural administration of 3 ml dexmedetomidine (0.5 μg kg^-1^) and then a continuous epidural administration of 80 μg dexmedetomidine in 150 ml levobupivacaine 0.125% at 3 ml h^-1^ for two days. Patients in the M group had the following treatment regimen: a loading dose epidural administration of 3 ml morphine (0.03 mg kg^-1^) and then a continuous epidural administration of 4.5 mg morphine in 150 ml levobupivacaine 0.125% at 3 ml h^-1^ for two days.

### Preparation for anesthesia

The basic pain threshold was expressed as the pain threshold (PTh) and pain tolerance threshold (PTTh), and the postoperative pain was measured using an 11-point verbal rating score (VRS, 0 to 10). All patients received midazolam 2.0 mg intravenously (IV) and fentanyl 0.05 mg IV five minutes before the baseline measurement and the epidural catheterization. Baseline measurements included heart rate (HR), non-invasive arterial blood pressure, respiratory rate, peripheral oxygen saturation, PTh, and PTTh.

### Implementation of anesthesia

The epidural catheter was placed in the T10/11 interspace using a midline approach. The epidural space was identified by loss of resistance to saline and a test-dose of 2% lignocaine with 1:200,000 adrenaline 3.0 ml to detect intrathecal or intravascular misplacement. After the test, 0.33% levobupivacaine was administered. When the sensory block level was ideal, patients underwent anaesthetic induction and tracheal intubation after propofol 2 mg kg^-1^, fentanyl 3μg kg^-1^ and vecuronium 0.1 mg kg^-1^, and general anesthesia was maintained with 2 L min^-1^ 50% O_2_ and 1.3~2.0% sevoflurane using a semi-closed circle system. Muscle relaxation was maintained with vecuronium 0.1 mg (kg h)^-1^. Patients received 5 ml levobupivacaine 0.33% at one hour intervals until the end of surgery. When the surgeon closed the peritoneum, a continuous infusor (Baxter®, USA) was attached to the epidural catheter for 48 h postoperative pain control.

### Assessments

To maintain blinding, the anaesthetist who prepared the study solution did not perform the epidural and was not involved in management or assessments. VRS was assessed at 2h, 4h, 6h, 8h, 16h, 24h and 48h after surgery, both at rest and after coughing. Postoperative analgesic requirements were met with IV flurbiprofen 100 mg at the patient’s request. The time to the first analgesic and total dose of analgesic were recorded. The times to postoperative first flatus (FFL) and first feces (FFE) were recorded, which were the primary and second efficacy endpoints of the study, respectively. When FFL occurred within the first postoperative 6 hours, we considered the occurrence of FFL may reflect the emptying of rectal gas rather than recovery of colonic transit, and the time to second flatus was recorded as the true recovery of colonic transit. Side effects potentially related to dexmedetomidine and morphine, such as bradycardia, hypotension, nausea and vomiting, skin itching were recorded. Hypotension was defined as the mean arterial pressure less than 30% from baseline for 60 seconds, and bradycardia was defined as heart rate (HR) less than 50 beats per minute. For the assessment of the safety of epidural dexmedetomidine, neurologic deficits included pain, numbness, and lack strength, and were assessed at 24 h, 48 h, 72h and 7 days after surgery.

### Statistical analysis

A power analysis was performed using the PASS 13.0 software (NCSS LLC) and a two-sample t-test was used based on a previous pilot study (means: 37.4 h (dexmedetomidine group) and 55.9 h (morphine group); SD: 22.8 h) revealed a sample size of 33 patients was required in each group to achieve a power of 90% and an α of 0.05 for detection of difference in the time to FFL between the two groups. To compensate for possible dropouts, a total 74 cases (37 for each group) were enrolled for study. Data were tested for normal distribution using the Kolmogorov-Smirnov test. Parametric data were described as mean and standard deviation, and non-parametric data as median and interquartile range. Gender, type of colectomy, and the incidence of complications were analysed using the chi-squared test. The Mann-Whitney U test was used to compare the pain scores between groups. For other variables showing a normal distribution, an independent two-sample t-test was used for intergroup comparisons. P <0.05 was considered statistically significant. All statistical analyses were performed using SPSS 19.0 software (IBM^®^, Armonk, NY, USA).

## Results

**Fig 1:**
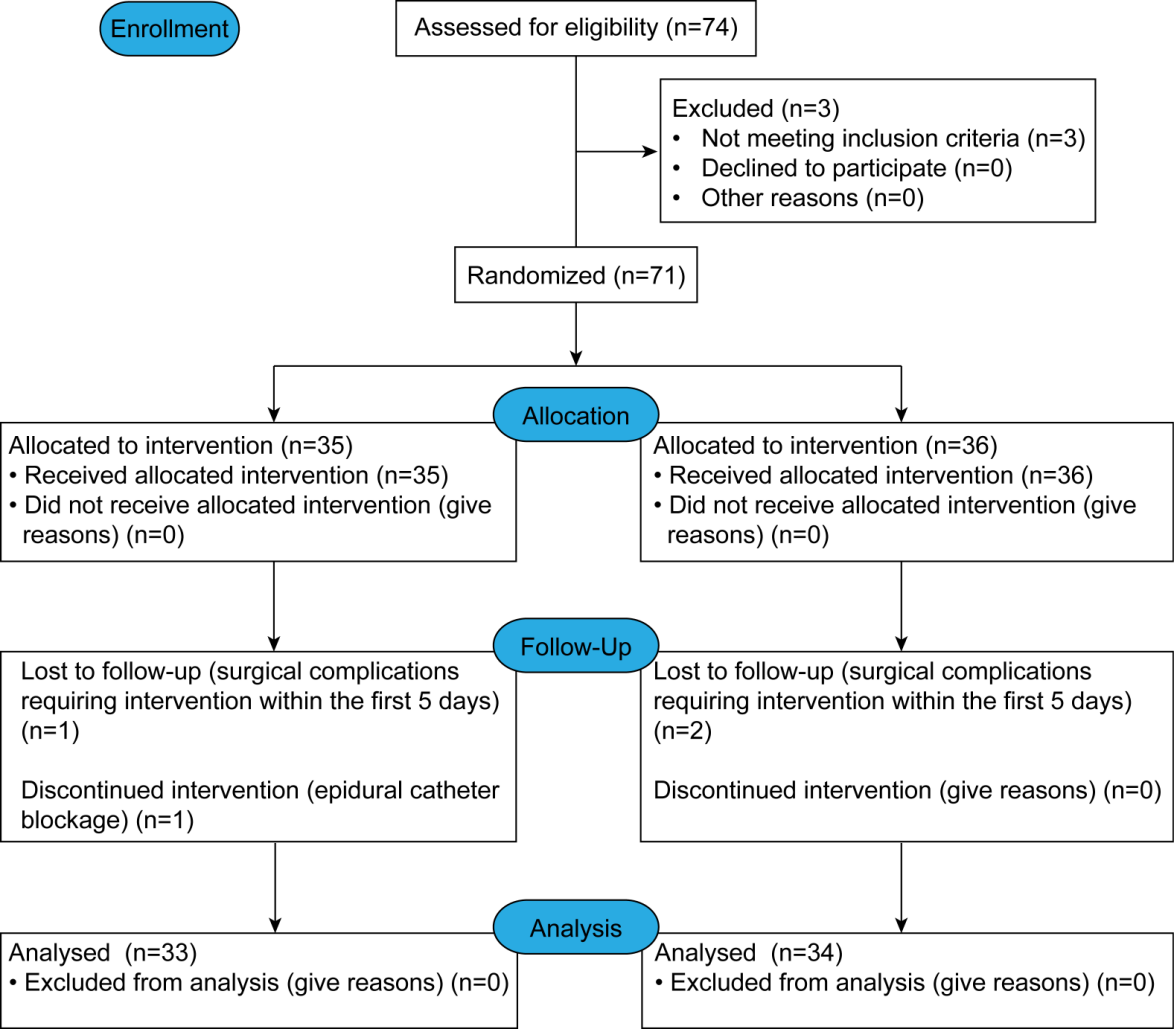
CONSORT 2010 flow diagram.

A total of 74 patients were randomly assigned to D or M treatment groups. Three patients (n = 1 from the D group; n = 2 from the M group) were withdrawn for various reasons: a history of gastrointestinal midline laparotomy (n = 2), and psychiatric disease (n = 1). The postoperative analgesic solution and gastrointestinal motility assessments were performed in 71 patients. Three of these had mechanical bowel obstructions or surgical complications requiring intervention within the first 5 days: 2 D group patients (internal herniation, n = 1; adhesion, n = 1); 1 M group patient (intraperitoneal bleeding, n = 1). One patient in the M group was excluded because of epidural catheter blockage. Therefore, 67 patients completed the study (Fig 1). Demographics and surgical aspects did not differ significantly between the two groups (Table 1).

**Table 1:** Baseline characteristics and surgical aspects of the included patients in both the groups.

	D group (n = 34)	M group (n = 33)	P
Age (year) ^a^	58 ± 11	61 ± 6	0.155 ^b^
Male Sex (%)	59	58	0.918 ^c^
Body mass index (Kg/m^2^) ^a^	24.1 ± 3.1	23.1 ± 2.8	0.23 ^b^
Type of colectomy (%)			0.54 ^c^
Right-sided	60	42	
Left-sided	14	21	
Sigmoid	26	37	
Duration of surgery (min) ^a^	184 ± 21	179 ± 18	0.298 ^b^
PTh (mA) ^a^	1.54 ± 0.1	1.53 ± 0.1	0.747 ^b^
PTTh (mA) ^a^	2.54 ± 0.2	2.51 ± 0.3	0.648 ^b^

^a^ Values are mean ± SD.

^b^ Independent two-sample t-test

^c^ Chi-squared test.

D = dexmedetomidine, M = morphine, PTh = pain threshold, PTTh = pain tolerance threshold.

### Postoperative pain control

At rest, the highest median VRS was 2 and the maximum range of interquartile range was 0 to 3 at all points of evaluation for both groups, indicating satisfactory levels of pain control. There were no significant differences between the M and D groups at any timepoint. During coughing, VRS increased at the majority of timepoints, however the highest median VRS scores was 3 and the maximum range of interquartile range was 0 to 4 at all points of evaluation for both groups, indicating a similar satisfactory level of pain control. In addition, no significant differences were observed between groups in either the time to the first analgesic dose or the total dose of analgesic (Table 2).

**Table 2:** Post-operative pain rating during 48 h, the time to the first analgesic and total dose of analgesic in both the groups.

	D group (n = 34)	M group (n = 33)	P
VRS (Rest/Cough) ^d^			
2h	0(0–0) / 1(0.75–3)	0(0–0) / 2(0–3)	0.62/0.54 ^e^
4h	1(0–1) / 2(1–3)	0(0–1) / 2(0–3)	0.482/0.067 ^e^
6h	1(0–1.25) / 2.5(2–3)	1(0–1.5) / 2(1–4)	0.659/0.618 ^e^
8h	1(1–1) / 3(2–3)	1(0–2) / 2(1–3)	0.632/0.217 ^e^
16h	2(0–3) / 2(2–4)	1(0.5–2) / 2(2–3)	0.458/0.568 ^e^
24h	1(1–2) / 2(2–4)	1(0–2) / 3(2–4)	0.607/0.803 ^e^
48h	1(1–2) / 2(2–3)	1(0–1.5) / 2(1–3)	0.084/0.125 ^e^
The time to the first analgesic ^a^	13.7 ± 7	15 ± 8.1	0.486 ^b^
The total dose of analgesic ^a^	129 ± 67.6	121 ± 65	0.615 ^b^

^a^ Values are mean ± SD.

^b^ Independent two-sample t-test

^d^ Values are median (interquartile range)

^e^ Mann-Whitney U test.

D = dexmedetomidine, M = morphine, VRS = verbal rating score.

### Recovery of gastrointestinal motility

**Fig 2:**
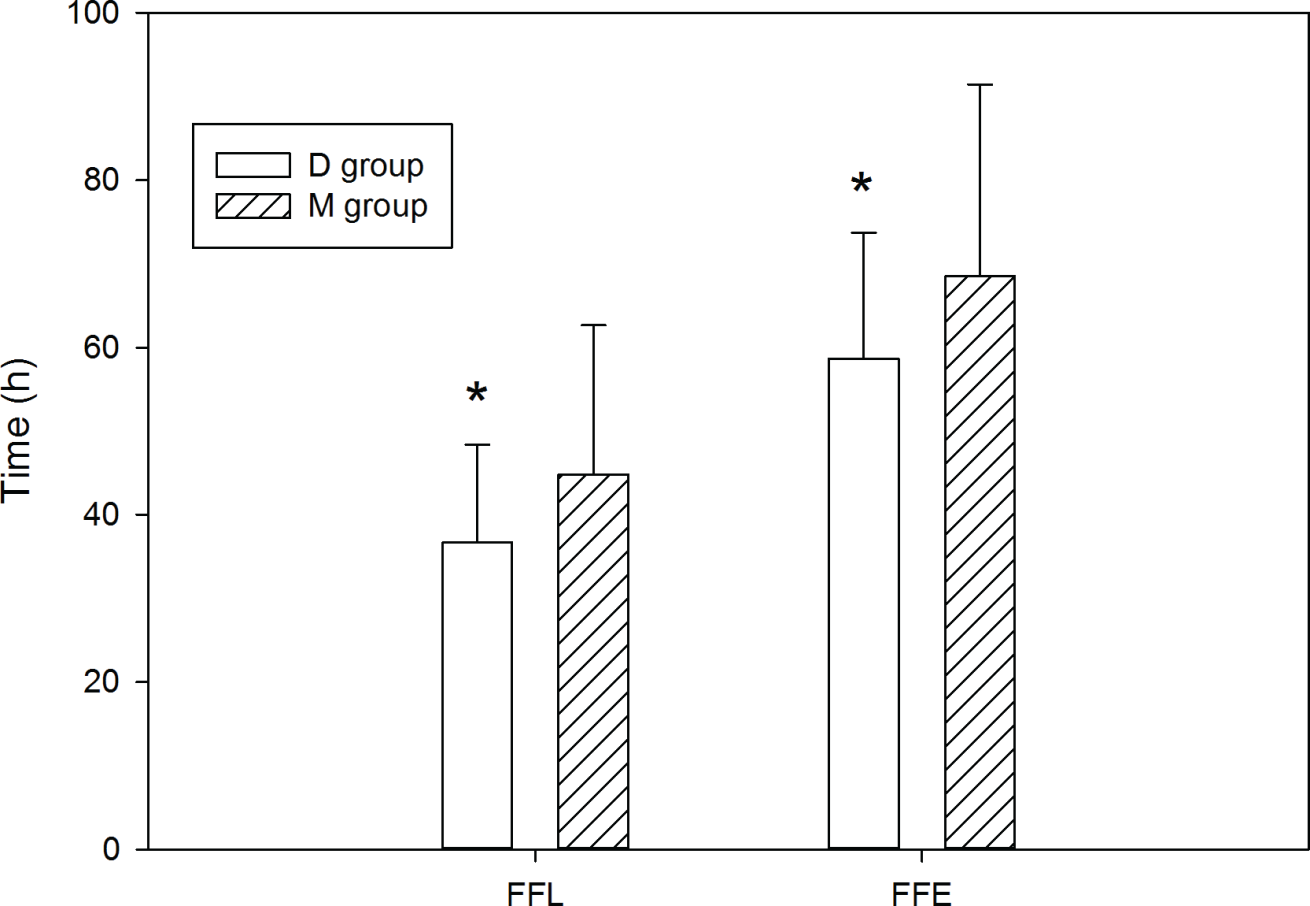
Time to postoperative first flatus and first feces of the two groups. Values are mean ± SD. FFL = first flatus, FFE = first feces, D = dexmedetomidine, M = morphine. * P<0.05 compared with M group.

The time to the FFL was 36.7 ± 11.7 h for the D group (mean ± SD), and 44.8 ± 17.8 h for the M group (*P* < 0.05). The time to the FFE was 58.7 ± 15 h for the D group and 68.5 ± 22.9 h for the M group (*P* < 0.05) (Fig 2). Thus, the D group demonstrated a significantly shorter time period for recovery of gastrointestinal motility, across both study endpoints.

### Postoperative side effects related analgesic

Patients in the M group had a higher incidence of nausea and vomiting and pruritus in comparison to the D group (Table 3, *P* < 0.05). In contrast, there were no significant differences between the M and D groups in the incidence of bradycardia or hypotension. No patients showed neurologic deficits in either group (Table 3).

**Table 3:** The comparison of postoperative side effects observed in both the groups.

Side effects (%)	D group (n = 34)	M group (n = 33)	P
Nausea and vomiting	15	33	0.042 ^c^
Skin itching	0	15	0.018 ^c^
Bradycardia	6	12	0.371 ^c^
Hypotension	9	12	0.659 ^c^
Neurologic deficits	0	0	

^c^ Chi-squared test.

D = dexmedetomidine, M = morphine.

## Discussion

First, our study found that patients who received low dose epidural dexmedetomidine in addition to levobupivacaine showed similar results for postoperative pain control in comparison to the co-administration of epidural morphine and levobupivacaine. Furthermore, gastrointestinal motility function was significantly improved with low dose epidural dexmedetomidine in comparison to epidural morphine. In our study, dexmedetomidine did not cause any discernable side effects. In fact, dexmedetomidine resulted in a reduced incidence of nausea, vomiting, and pruritus in comparison to epidural morphine.

The results of postoperative pain control in this study indicate that co-administration of low dose epidural dexmedetomidine and levobupivacaine can provide approximate analgesia compared with morphine and levobupivacaine. The mechanisms by which dexmedetomidine mediates intra-epidural analgesia are still not clear, but may be related to two aspects. Firstly, dexmedetomidine is known to improve the analgesia effect of local anaesthetics. For example, dexmedetomidine binds α2A, α2B and α2C adrenoceptors with high affinity.[12] Tatsushi Y *et al* found that the α2-adrenoceptor agonist dose dependently enhanced local anesthetic action via α2A-adrenoceptors.[13] Thus, the analgesic effect mediated by epidural dexmedetomidine may reflect synergy with levobupivacaine. On the other hand, epidural dexmedetomidine itself may provide analgesia. Due to its lipophilicity, dexmedetomidine rapidly appears in the cerebrospinal fluid and has been demonstrated to exhibit a central analgesic effect.[3, 14] In addition, the analgesic effects may be mediated through spinal, supraspinal, and peripheral action. Experts have found that adrenergic agonists can inhibit release of peptides from spinal cord slices and inhibit dorsal horn nociceptive neurons.[15] Moreover, a recent study indicated that the antinociceptive effect of dexmedetomidine may result from its action on spinal cord substantia gelatinosa.[16] In summary, dexmedetomidine may exert both direct and indirect analgesic effects, although the precise mechanisms are only partly understood.

The present study found that low dose epidural dexmedetomidine significantly shortens the time to postoperative first flatus and feces, consistent with our prior observations.[8] However and as previously mentioned, some disagreement exists in the literature regarding the precise effects of dexmedetomidine on GI motility. For example, one previous study assessed the gastrointestinal effects of dexmedetomidine infusion in humans and observed that dexmedetomidine did not significantly inhibit gastric emptying.[17] On the other side, another animal study showed that dexmedetomidine weakly inhibited gastric emptying.[11] Although overall dexmedetomidine increased intestinal transit time, this increase was less potent than morphine.[11] The slight contradictions in the literature on the subject of dexmedetomidine effects on gastrointestinal motility may be easily explained by differences in dosage and route of delivery. For example, investigators found that the range of ED_50_ (50% Effective Dose) for the antinociceptive effect of dexmedetomidine (which was systemic administered) is much less than the ED_50_ for its effects on gastric emptying. In contrast, the ED_50_ for the antinociceptive effect of morphine includes the ED_50_ for gastric emptying.[11] Overall, these results suggest that the antinociceptive effects of dexmedetomidine may be achieved without detrimental effects on GI motility. These results also further support the beneficial effects of dexmedetomidine on gastrointestinal motility, as compared to morphine.

Importantly, the dose of dexmedetomidine may also contribute to the gastrointestinal motility effects. Two clinical studies with opposite results demonstrate the dose dependent effects of dexmedetomidine. Patients received dexmedetomidine 1.0 μg kg^-1^ infused over 20 min, followed by a continuous infusion of 0.7μg (kg h) ^-1^ for 190 min (total dose nearly 3.1 μg kg^-1^) showed inhibited gastric emptying and gastrointestinal transit.[10] In contrast, gastric emptying was not delayed when the total dose was decreased to 1.0 μg kg^-1^.[17]

With respect to safety issues, only levobupivacaine, dexmedetomidine and sodium chloride, without preservatives, were administered epidurally in our study. One animal experiment found that epidural dexmedetomidine may have harmful effects on the myelin sheath, possibly related to the pH of dexmedetomidine.[18] Anaesthetic solutions of low pH were once suspected to cause neurotoxicity, but this notion has been subsequently discredited.[19] Importantly, no patients in our study showed neurologic deficits. This result was consistent with previous studies, and suggested that this epidural anaesthetic management for postoperative pain control is safe. Importantly, our study is limited by a relatively modest sample size, and future studies must confirm the safety of epidural dexmedetomidine. Many studies have used peripheral, intrathecal or epidural injection without reporting neurologic deficits,[4–8, 20–23] with some of these studies administering higher epidural doses than used in the present study. Animal experiments support the safety of intrathecal dexmedetomidine as well. For example, dexmedetomidine showed no significant pathologic impact on the spinal cord, and in fact showed potential protective effects against lignocaine-induced neural cell death[24]. Thus, while large scale studies are needed, epidural dexmedetomidine appears to be both safe and efficacious.

There are several limitations to the present study. All the patients in this study were ASA I or II and without significant co-morbidities. Therefore, the result of this study cannot be generalized for ASA III patients and above. Also, the parameters used to evaluate gastrointestinal motility were clinical, whereas scintigraphic recording of gastrointestinal transit is considered the gold standard. In future studies, we will confirm the results using scintigraphic recording and extend our research to include ASA III patients and above. In conclusion, the present clinical study highlights the benefit of epidural dexmedetomidine as an adjunct for postoperative pain control with levobupivacaine.

## Supporting Information

S1 CONSORT Checklist CONSORT 2010 checklist. (DOC)

S1 Protocol Protocol in English. (DOC)

S2 Protocol Protocol in Chinese. (DOC)
