# Expression of Concern: Epidural Co-Administration of Dexmedetomidine and Levobupivacaine Improves the Gastrointestinal Motility Function after Colonic Resection in Comparison to Co-Administration of Morphine and Levobupivacaine

**DOI:** 10.1371/journal.pone.0279703

**Published:** 2022-12-21

**Authors:** The PLOS ONE Editors

Following publication of this article [1], concerns were raised about the reported methodology and about potential discrepancies between the trial documentation and the information reported in the article. The *PLOS ONE* Editors reviewed the ethical approval documentation and followed up on the issues raised with the authors and their institution to clarify the following issues:

- The Institutional Research Board (IRB) approval document reports a study program start date that precedes the ethical approval date. The corresponding author indicated that the planned start date changed and that the patient enrollment dates reported in the Methods section of the article are correct. A representative of the Harbin Medical University Ethics Review Committee stated that the study commenced after the ethical approval date of 3 March, 2014.
- The Clinical Trial Registration (ChiCTR-TRC-14004644) mentions three treatment groups (M, L, and D), while the published article [1] reports on two treatment groups (M and D). The IRB approval document outlines methodology for two groups, but includes a Conclusions/Significance section that appears to report findings from three treatment groups. The corresponding author indicated that the initial study design included three treatment groups, but that the “L” group (levobupivacaine alone) was removed in response to the ethical review, and that the Conclusions/Significance section of the IRB approval document describes the expected research results at the time of the initial ethics application. A representative of the Harbin Medical University Ethics Review Committee stated that the researchers conducted the experiments according to the ethically approved protocol.
- Reference [8] in the original article [1] is described as a “pilot study” in which “epidural dexmedetomidine…shortened the time of first flatus of patients after nephrectomy” though this was noted as a trend that did not reach statistical significance. The corresponding author indicated that time to first flatus data were observed but not published in reference [8] and that reference [8] was not the pilot study for [1]. The corresponding author indicated that a separate pilot study of 35 patients (17 in group D, 18 in group M) was carried out prior to the main study reported in [1], which included 75 patients. The corresponding author also indicated that both the pilot study (n = 35) and the main study (n = 75) were carried out under the IRB approval HMUIRB20140004. However, it is noted that the English language approval document specifies only a single study of 75 patients, while the Chinese language version of the document does not specify a sample size.
- The IRB approval document indicates that group D is approved to receive a dose of 60μg dexmedetomidine in 150ml levobupivocaine 0.125% at 3ml h^-1^ for two days. The article reports the use of a higher dosage of 80μg dexmedetomidine in 150ml levobupivocaine 0.125% at 3ml h^-1^ for two days. The corresponding author indicated that the initial study design used a lower dose of epidural dexmedetomidine in consideration of safety, but that it was later found that 60μg dexmedetomidine could not provide a satisfactory analgesic effect, and after discussion with experts it was considered that the dosage should be increased to 80 μg, but it was not reported to the ethics committee in time to revise the dose. The higher dose of 80μg dexmedetomidine was used for both the pilot study and the main study. A representative of the Harbin Medical University Ethics Review Committee stated that the modified dose was not reported to the ethics committee in time.

Given the information received in post-publication follow up, the *PLOS ONE* Editors consider that the concerns about discrepancies in the study start dates and the study design are resolved. The *PLOS ONE* Editors issue this Expression of Concern in light of the divergence from the IRB-approved dosage that was not reported to the IRB in advance of the pilot study or the main study, and further note that it is not clear from the available documentation that the ethical approval covered an additional pilot study of 35 patients.

The authors have provided the underlying data for the study as Supporting Information (S1 File). The data were reviewed by a member of the Editorial Board, who identified errors in Tables 1–3 and noted that the Kolmogorov-Smirnov test used to test data for normal distribution is an acceptable test but has low power with small sample sizes.

**Table 1:** Baseline characteristics and surgical aspects of the included patients in both the groups.

	D group (n = 34)	M group (n = 33)	P
Age (year) ^a^	58 ± 11	61 ± 6	0.155 ^b^
Male Sex (%)	59	58	0.918 ^c^
Body mass index (Kg/m^2^) ^a^	24.1 ± 3.1	23.1 ± 2.8	0.23 ^b^
Type of colectomy (%)			0.405 ^c^
Right-sided	59	42	
Left-sided	15	21	
Sigmoid	26	37	
Duration of surgery (min) ^a^	184 ± 21	179 ± 18	0.298 ^b^
PTh (mA) ^a^	1.54 ± 0.1	1.53 ± 0.1	0.747 ^b^
PTTh (mA) ^a^	2.54 ± 0.2	2.51 ± 0.3	0.648 ^b^

^a^ Values are mean ± SD.

^b^ Independent two-sample t-test

^c^ Chi-squared test.

D = dexmedetomidine, M = morphine, PTh = pain threshold, PTTh = pain tolerance threshold.

**Table 2:** Post-operative pain rating during 48 h, the time to the first analgesic and total dose of analgesic in both the groups.

	D group (n = 34)	M group (n = 33)	P
VRS (Rest/Cough) ^d^			
2h	0(0–0) / 1(0.75–3)	0(0–0) / 2(0–3)	0.62/0.54 ^e^
4h	1(0–1) / 2(1–3)	0(0–1) / 2(0–3)	0.482/0.067 ^e^
6h	1(0–1.25) / 2.5(2–3)	1(0–1.5) / 2(1–4)	0.659/0.618 ^e^
8h	1(1–1) / 3(2–3)	1(0–2) / 2(1–3)	0.632/0.217 ^e^
16h	2(0–3) / 2(2–4)	1(0.5–2) / 2(2–3)	0.458/0.568^e^
24h	1(1–2) / 2(2–4)	1(0–2) / 3(2–4)	0.607/0.803 ^e^
48h	1(1–2) / 2(2–3)	1(0–1.5) / 2(1–3)	0.084/0.125 ^e^
The time to the first analgesic ^a^	13.7 ± 7	15 ± 8.1	0.486 ^b^
The total dose of analgesic ^d^	100(100–200)	100(100–200)	0.679 ^e^

^a^ Values are mean ± SD.

^d^ Values are median (interquartile range)

^b^ Independent two-sample t-test

^e^ Mann-Whitney U test.

D = dexmedetomidine, M = morphine, VRS = verbal rating score.

**Table 3:** The comparison of postoperative side effects observed in both the groups.

Side effects (%)	D group (n = 34)	M group (n = 33)	P ^c^
Nausea and vomiting	15	36	0.042
Skin itching	0	15	0.018
Bradycardia	6	12	0.371
Hypotension	9	12	0.659
Neurologic deficits	0	0	

^c^ Chi-squared test.

D = dexmedetomidine, M = morphine.

In Table 1, there is an error in the percentage of right-sided versus left-sided colectomies, and in the p-value for type of colectomy. Here the authors provide a revised Table 1.

In Table 2, the underlying data for total dose of analgesic are not normally distributed, so the independent two-sample t-test is not appropriate. Here the authors provide a revised Table 2 in which the values are reported as mean (interquartile range) and the p-value is calculated using the Mann-Whitney U test.

In Table 3, there is an error in the percentage of patients reported as experiencing nausea and vomiting. Here the authors provide a revised Table 3.

The corresponding author stated that the revised results still support the conclusions of the article.

In the published article [1], the fifth paragraph of the Discussion section includes text that was previously published in an earlier study by some of the same authors (cited as reference [8] of the article).

## Supporting information

S1 File Data. (XLSX)
