# Correction: Expression of Concern: Epidural co-administration of Dexmedetomidine and Levobupivacaine improves the Gastrointestinal motility function after colonic resection in comparison to co-administration of Morphine and Levobupivacaine

**DOI:** 10.1371/journal.pone.0340227

**Published:** 2026-01-20

**Authors:** The PLOS One Editors

## Notice of Republication

This Expression of Concern [1] was republished on December 17, 2025, to address an issue identified post-publication and provide an updated version of the Supporting Information. Please download this article again to view the correct version.

## References

[1] The *PLOS ONE* Editors. Expression of concern: Epidural co-administration of Dexmedetomidine and Levobupivacaine improves the Gastrointestinal motility function after colonic resection in comparison to co-administration of Morphine and Levobupivacaine. PLoS One. 2022;17(12):e0279703. doi: 10.1371/journal.pone.0279703 36542652 PMC9770331

