# Correction: Epidural Co-Administration of Dexmedetomidine and Levobupivacaine Improves the Gastrointestinal Motility Function after Colonic Resection in Comparison to Co-Administration of Morphine and Levobupivacaine

**DOI:** 10.1371/journal.pone.0340228

**Published:** 2026-01-20

**Authors:** The PLOS One Editors

## Notice of Republication

This article [1] was republished on December 17, 2025, to address an issue identified post-publication. Please download this article again to view the correct version.

## References

[1] Zeng X-Z, Lu Z-F, Lv X-Q, Guo Y-P, Cui X-G. Epidural Co-Administration of Dexmedetomidine and Levobupivacaine Improves the Gastrointestinal Motility Function after Colonic Resection in Comparison to Co-Administration of Morphine and Levobupivacaine. PLoS One. 2016;11(1):e0146215. doi: 10.1371/journal.pone.0146215 26751791 PMC4709108

