# Supplementary material for: Epidural Co-Administration of Dexmedetomidine and Levobupivacaine Improves the Gastrointestinal Motility Function after Colonic Resection in Comparison to Co-Administration of Morphine and Levobupivacaine
Source: PLoS One. 2016 Jan 11;11(1):e0146215. doi: 10.1371/journal.pone.0146215 (PMC4709108; doi:10.1371/journal.pone.0146215)
Supplement: S1 Protocol — (DOC) [file pone.0146215.s002.doc]

**SECOND HOSPITAL OF HARBIN MEDICAL UNIVERSITY**

**Title：**The Effect of Epidural Dexmedetomidine on Intestinal Peristalsis Function After Colonic Resection

# **Applicant：**Xiaoguang Cui

**Department：**Department of Anesthesiology

**TEL：** 0451-86605452

**E-mail： cuixiaoguang1018@126.com**

**Starting and ending time：**2013～2014

**Second Hospital of Harbin Medical University**

**March 2013**

**1、Basic information**

| **申**  **请**  **人**  **信**  **息** | **Name** | Xiaoguang Cui | **Sex** | **male** | **Year of birth** | 1966 years january |
| --- | --- | --- | --- | --- | --- | --- |
|  | **Academic degree** | doctor | professional post | **professor** | **Research field** | Clinical anesthesia |
| **课题基本信息** | **项目名称** | **中 文** | 硬膜外使用右美托咪定对结肠手术病人肠蠕动功能的影响 | | | |
|  |  | **英 文** | The effect of epidural dexmedetomidine on the intestinal peristalsis function after colonic resection | | | |
|  | **中文关键词** | 右美托咪定，硬膜外麻醉，肠运动 | | | **英文关键词** | dexmedetomidine，epidural anesthesia, intestinal peristalsis |

**2、Brief project introduction**

The objective of the present study is to investigate the effects of low dose epidural dexmedetomidine on gastrointestinal motility after colonic resection in comparison to the co-administration of morphine and levobupivacaine. Gastrointestinal motility is commonly impaired after intestinal surgery, which causes an increased incidence of postoperative ileus. Co-administration of epidural morphine and local anaesthetic is a common and effective method of postoperative pain control. However, postoperative epidural morphine commonly known to further reduce gastrointestinal motility. Many studies have found that dexmedetomidine may induce both central and peripheral analgesic effects. Aditionally, our previous studies found that epidural dexmedetomidine not only significantly potentiated the effects of local anesthetics, but also shortened the time of first flatus after nephrectomy. We hypothesized that dexmedetomidine influences gastrointestinal motility, and speculated that differences in the dose and route of administration of dexmedetomidine may largely explain the contradictory reports present within the literature.

**3、Other researchers**

| **序号** | **姓名** | **出生**  **年月** | **性 别** | **职称/职务** | **学位** | **所在科室** | **任务分工** | **签字** |
| --- | --- | --- | --- | --- | --- | --- | --- | --- |
| 1 | Xianzhang Zeng |  | 男 | 主治医师 | 硕士 | 麻醉科 | 实施麻醉，收集数据，攥写论文 |  |
| **2** | Zhifang Lu |  | 男 | 医师 | 本科 | 麻醉科 | 实施麻醉 |  |
| **3** | Xiangqi Lv |  | 女 | 副主任医师 | 博士 | 麻醉科 | 数据统计分析 |  |
| **4** | Yueping Guo |  | 男 | 副主任医师 | 博士 | 麻醉科 | 实施麻醉 |  |
| 5 | Xiaoguang Cui |  | 男 | 主任医师 | 博士 | 麻醉科 | 实验设计管理 |  |

## **4、Project basis**

| Gastrointestinal motility is commonly impaired after intestinal surgery, which causes an increased incidence of postoperative ileus. Co-administration of epidural morphine and local anesthetic is a common and effective method of postoperative pain control. However, postoperative epidural morphine has been widely found to further reduce gastrointestinal motility. An optimal epidural anesthetic for postoperative pain management that does not impair gastrointestinal motility is needed.  Dexmedetomidine is a centrally selective α2-adrenoceptor agonist that can be given as either a sedative or an analgesic without significant respiratory depression. In recent years, dexmedetomidine has been administered perineurally to block nerve conduction and as a neuraxial analgesia for spinal or epidural anaesthesia in combination with local anaesthetics. In these studies, dexmedetomidine potentiated anaesthesia with few side effects. In our previous study, epidural dexmedetomidine not only significantly potentiated the effects of local anesthetics, but also shortened the time of patients’ first flatus after nephrectomy. Although statistical significance was not observed in this pilot study, the trend was compelling and warranted further studies on a larger scale. The hypothesis that epidural morphine, also given in the prior study, may have inadvertently masked the beneficial effects of epidural dexmedetomidine is supported by animal studies. For example, investigators have found that dexmedetomidine alone showed beneficial effects on the recovery of intestinal motility in rats. However, some disagreement in the literature still exists regarding the effects of dexmedetomidine on gastrointestinal function. For example, a clinical study showed that dexmedetomidine inhibits gastric emptying and gastrointestinal transit time in healthy volunteers who had not undergone surgery. Furthermore, investigators also found that dexmedetomidine can inhibit intestinal transit in rats, although this inhibition was less potent than with morphine. Thus, although some studies suggest that dexmedetomidine is preferential to morphine for gastrointestinal motility, the benefits of epidural dexmedetomidine remain largely unknown,.  From these studies, we hypothesized that dexmedetomidine influences gastrointestinal motility and speculated that differences in the dose and route of administration may largely explain the contradictory reports present within the literature. The objective of the present study is to investigate the effects of low dose epidural dexmedetomidine on gastrointestinal motility after colonic resection in comparison to the co-administration of morphine and levobupivacaine.    **References**  1 Fukuda H, Tsuchida D, Koda K, Miyazaki M, Pappas TN, Takahashi T. Impaired gastric motor activity after abdominal surgery in rats. Neurogastroenterol Motil 2005; 17: 245-50  2 Holte K, Kehlet H. Postoperative ileus: a preventable event. Br J Surg 2000; 87: 1480-93  3 Bonnet F, Vesinet C. [How can we improve the efficacy of morphine analgesia without increasing adverse effects?]. Cah Anesthesiol 1994; 42: 191-4  4 Thorn SE, Wattwil M, Naslund I. Postoperative epidural morphine, but not epidural bupivacaine, delays gastric emptying on the first day after cholecystectomy. Reg Anesth 1992; 17: 91-4  5 Gillan MG, Pollock D. Acute effects of morphine and opioid peptides on the motility and responses of rat colon to electrical stimulation. Br J Pharmacol 1980; 68: 381-92  6 Porreca F, Filla A, Burks TF. Spinal cord-mediated opiate effects on gastrointestinal transit in mice. Eur J Pharmacol 1982; 86: 135-6  7 Ebert TJ, Hall JE, Barney JA, Uhrich TD, Colinco MD. The effects of increasing plasma concentrations of dexmedetomidine in humans. Anesthesiology 2000; 93: 382-94  8 Esmaoglu A, Yegenoglu F, Akin A, Turk CY. Dexmedetomidine added to levobupivacaine prolongs axillary brachial plexus block. Anesth Analg 2010; 111: 1548-51  9 Rancourt MP, Albert NT, Cote M, Letourneau DR, Bernard PM. Posterior tibial nerve sensory blockade duration prolonged by adding dexmedetomidine to ropivacaine. Anesth Analg 2012; 115: 958-62  10 Salgado PF, Sabbag AT, Silva PC, et al. [Synergistic effect between dexmedetomidine and 0.75% ropivacaine in epidural anesthesia]. Rev Assoc Med Bras 2008; 54: 110-5  11 El-Hennawy AM, Abd-Elwahab AM, Abd-Elmaksoud AM, El-Ozairy HS, Boulis SR. Addition of clonidine or dexmedetomidine to bupivacaine prolongs caudal analgesia in children. Br J Anaesth 2009; 103: 268-74  12 Kanazi GE, Aouad MT, Jabbour-Khoury SI, et al. Effect of low-dose dexmedetomidine or clonidine on the characteristics of bupivacaine spinal block. Acta Anaesthesiol Scand 2006; 50: 222-7  13 Mohamed AA, Fares KM, Mohamed SA. Efficacy of intrathecally administered dexmedetomidine versus dexmedetomidine with fentanyl in patients undergoing major abdominal cancer surgery. Pain Physician 2012; 15: 339-48  14 Zeng XZ, Xu YM, Cui XG, Guo YP, Li WZ. Low-dose epidural dexmedetomidine improves thoracic epidural anaesthesia for nephrectomy. Anaesth Intensive Care 2014; 42: 185-90  15 Aydin C, Bagcivan I, Gursoy S, Altun A, Topcu O, Koyuncu A. Altered spontaneous contractions of the ileum by anesthetic agents in rats exposed to peritonitis. World J Gastroenterol 2009; 15: 1620-4  16 Iirola T, Vilo S, Aantaa R, et al. Dexmedetomidine inhibits gastric emptying and oro-caecal transit in healthy volunteers. Br J Anaesth 2011; 106: 522-7  17 Asai T, Mapleson WW, Power I. Differential effects of clonidine and dexmedetomidine on gastric emptying and gastrointestinal transit in the rat. Br J Anaesth 1997; 78: 301-7 |
| --- |

**5、Research objectives**

| To improve the gastrointestinal motility function of colonic resection patients using epidurally administered dexmedetomidine. |
| --- |

**6、Study design**

| Methods  With written consent, 74 American Society of Anaesthesiology Physical Status I/II patients undergoing elective colonic resection were enrolled. To eliminate any discrepencies due to differences in surgical technique, all procedures were undertaken by a single surgeon. The surgeries were performed through a midline laparotomy and all anastomoses were performed using mechanical staplers. Patients received standardized care during the perioperative period and were allowed to intake small amounts of water orally during the first 24h post-surgery and semi-solid food thereafter. A normal diet was not allowed until after the first flatus.  The exclusion criteria were: neurologic or psychiatric disease, a known allergy to local anaesthetic agents, diabetes, a history of gastrointestinal motility disorders or prior abdominal surgery, renal or hepatic insufficiency, bleeding or coagulation abnormalities, or anticoagulant therapy.  Using a computer-generated random number table, the patients were randomly assigned to one of two groups and received different postoperative analgesia plans when the surgeon closed the peritoneum. If a patient was allocated an odd number, (s)he was placed in the dexmedetomidine group (D group). If a patient was allocated an even number, (s)he was placed in the morphine group (M group). D group: patients received a loading dose of 3 ml dexmedetomidine (0.5 μg kg^-1^), administered epidurally, and then a continuous epidural administration of 80 μg dexmedetomidine in 150 ml levobupivacaine 0.125% at 3 ml h^-1^ for two days. M group: patients received a loading dose of 3 ml morphine (0.03 mg kg^-1^), administered epidurally, and then a continuous epidural administration of 4.5 mg morphine in 150 ml levobupivacaine 0.125% at 3 ml h^-1^ for two days.  **Experimental steps:**  1.The baseline pain threshold was expressed as the pain threshold (PTh) and pain tolerance threshold (PTTh), and the postoperative pain was measured using an 11-point verbal rating score (VRS, 0 to 10). The method of measuring PTh and PTTh, and the instructions for using the VRS were explained to all patients. PTh and PTTh were measured using the microcurrent stimulation method by a PTh detector (HD-EP-601C detector; Hengaode instrument company, Beijing, China) on the left upper arm. The detector generates a 50-Hz electrical stimulation with 0.5-ms pulse width. The intensity was increased gradually at the rate of 0.1 mA/s from 0 to 5 mA. The electrical PTh was reached when the patient first felt pain and PTTh was reached when the patient felt that the pain was intolerable. The test was carried out again after 10 min and the average value of the two tests was calculated and recorded.  2. All patients received midazolam 2.0 mg intravenously (IV) and fentanyl 0.05 mg IV five minutes before the baseline measurement and the epidural catheterization. Baseline measurements included heart rate (HR), non-invasive arterial blood pressure, respiratory rate, peripheral oxygen saturation, PTh, and PTTh.  3. The epidural catheter was placed in the T10/11 interspace using a midline approach. The epidural space was identified by loss of resistance to saline and a test-dose of 2% lignocaine with 1:200,000 adrenaline. 3.0 ml was administered to detect intrathecal or intravascular misplacement. After the test, 0.33% levobupivacaine was administered through the epidural catheter and patients underwent anaesthetic induction and tracheal intubation after receiving 2 mg kg^-1^ ,propofol, 3μg kg^-1^ fentanyl, and 0.1 mg kg^-1^ vecuronium. General anaesthesia was maintained with 50% O_2_ at 2 L min^-1^ and 1.3~2.0% sevoflurane using a semi-closed circuit system. Muscle relaxation was maintained with using 0.1 mg (kg h)^-1^ vecuronium.. Patients received 5 ml 0.33% levobupivacaine at one hour intervals until the end of surgery. A central venous catheter was placed through into the right subclavian vein for perioperative infusion and postoperative intravenous nutrition, as commonly performed at our institution. When the surgeon closed the peritoneum, a continuous infusor (Baxter®, USA) was attached to the epidural catheter for 48 h postoperative pain control.  **Data Aquisiton:**  To maintain blinding, the anesthesiologist who prepared the solutions did not perform the epidural and was not involved in management or assessments. VRS was assessed at 2h, 4h, 6h, 8h, 16h, 24h and 48h after surgery, both at rest and after coughing. Postoperative analgesic requirements were met with 100 mg IV flurbiprofen upon patient request. Time to the first analgesic and total dose of analgesic were recorded. Time to first flatus (FFL) and first feces (FFE) were recorded, which served as the primary and secondary efficacy endpoints of the study. When the FFL occurred within the first 6 hours postoperative, we considered the occurrence of FFL may reflect the emptying of rectal gas rather than recovery of colonic transit, and the time to second flatus was recorded as the true recovery of colonic transit. The side effects related to dexmedetomidine and morphine, such as bradycardia, hypotension, nausea and vomiting, and skin itching were recorded. Hypotension was defined as a mean arterial pressure of less than 30% below baseline for 60 seconds, and bradycardia was defined as a heart rate (HR) of less than 50 beats per minute. For the assessment of the safety of epidural dexmedetomidine, neurologic deficits assessed included pain, numbness, and lack strength, and were assessed at 24 h, 48 h, 72h and 7 days after surgery. |
| --- |
| **（二）**Technology Roadmap  **Randomly**  （elective clonic resection, n=74）  D group： loading dose epidural administration of 3 ml dexmedetomidine (0.5 μg kg^-1^) and then a continuous epidural administration of 80 μg dexmedetomidine in 150 ml levobupivacaine 0.125% at 3 ml h^-1^ for two days  **Recorded**  VRS at 2h, 4h, 6h, 8h, 16h, 24h and 48h after surgery  The time to postoperative first flatus and first feces  The incidence of bradycardia, hypotension, nausea and vomiting, skin itching  M group：loading dose epidural administration of 3 ml morphine (0.03 mg kg^-1^) and then a continuous epidural administration of 4.5 mg morphine in 150 ml levobupivacaine 0.125% at 3 ml h^-1^ for two days. |
| **（三）Research Difficulties**    Randomization |

**七、Research Ethics**

| 1. Clinical commonly used drugs  2. Informed Consent |
| --- |
