# Supplementary material for: Epidural Co-Administration of Dexmedetomidine and Levobupivacaine Improves the Gastrointestinal Motility Function after Colonic Resection in Comparison to Co-Administration of Morphine and Levobupivacaine
Source: PLoS One. 2016 Jan 11;11(1):e0146215. doi: 10.1371/journal.pone.0146215 (PMC4709108; doi:10.1371/journal.pone.0146215)
Supplement: S2 Protocol — (DOC) [file pone.0146215.s003.doc]

**哈尔滨医科大学附属第二医院**

**伦理申请书**

# **课题名称：**硬膜外使用右美托咪定对结肠手术病人肠蠕动功能的影响

# **申 请 人： 崔晓光**

**所在科室： 麻醉科**

**联系电话： 0451-86605452**

**电子信箱： cuixiaoguang1018@126.com**

**起止年限：2013年～2014年**

**哈尔滨医科大学附属第二医院**

**二〇一三年三月制**

**一、基本信息**

| **申**  **请**  **人**  **信**  **息** | **姓名** | 崔晓光 | **性别** | 男 | **出生年月** | 1966年01月16日 |
| --- | --- | --- | --- | --- | --- | --- |
|  | **学位** | 博士 | **职称** | 主任医师 | **主要研究领域** | 临床麻醉 |
| **课题基本信息** | **项目名称** | **中 文** | 硬膜外使用右美托咪定对结肠手术病人肠蠕动功能的影响 | | | |
|  |  | **英 文** | The effect of epidural dexmedetomidine on the intestinal peristalsis function after colonic resection | | | |
|  | **中文关键词** | 右美托咪定，硬膜外麻醉，肠运动 | | | **英文关键词** | dexmedetomidine，epidural anesthesia, intestinal peristalsis |

二、课题内容简介（400字以内）

| 本研究探索一种与吗啡相比，促进结肠手术术后胃肠运动功能恢复的新的硬膜外镇痛用药方案。肠道手术后胃肠运动功能会受到损伤，从而导致术后肠梗阻等并发症的增加。目前世界范围内使用比较广泛的硬膜外镇痛方案是吗啡与局麻药混合，然而吗啡的使用会进一步抑制胃肠运动功能。  本课题组前期研究发现，硬膜外使用右美托咪定可能通过作用于中枢以及脊髓阶段的α2受体发挥中枢性及外周性镇痛作用。同时与硬膜外应用吗啡相比，可能对胃肠运动功能具有一定的益处。本研究的创新点在于首次将右美托咪定与局麻药混合用于胃肠手术的硬膜外镇痛，同时观察其与吗啡相比，对胃肠功能恢复的影响。 |
| --- |

**三、课题组人员基本情况（不包括申请人）**

| **序号** | **姓名** | **出生**  **年月** | **性 别** | **职称/职务** | **学位** | **所在科室** | **任务分工** | **签字** |
| --- | --- | --- | --- | --- | --- | --- | --- | --- |
| 1 | 曾宪章 |  | 男 | 主治医师 | 硕士 | 麻醉科 | 实施麻醉，收集数据，攥写论文 |  |
| **2** | 卢志方 |  | 男 | 医师 | 本科 | 麻醉科 | 实施麻醉 |  |
| **3** | 吕湘琪 |  | 女 | 副主任医师 | 博士 | 麻醉科 | 数据统计分析 |  |
| **4** | 郭悦平 |  | 男 | 副主任医师 | 博士 | 麻醉科 | 实施麻醉 |  |
| 5 | 崔晓光 |  | 男 | 主任医师 | 博士 | 麻醉科 | 实验设计管理 |  |

## **四、立题依据（**主要描述研究意义，包括研究涉及的相关健康问题是否严重？为什么该问题会长期存在？目前该领域现有的知识技术水平？该研究项目是否能产生出比现有技术手段更好的干预措施？国内外研究现状及发展动态分析，请附近五年主要参考文献）

| 肠道手术后胃肠运动功能会受到损伤，从而导致术后肠梗阻发生率增加。硬膜外使用吗啡与局麻药混合进行硬膜外镇痛是一种非常有效且应用广泛的术后镇痛方法。然而吗啡的使用会进一步抑制已经受损的胃肠运动功能。探索一种更为理想的硬膜外镇痛用药方案具有一定的临床意义。  右美托咪定是一种中枢性α2受体激动剂，近年来研究发现，椎管内使用右美托咪定是安全的，并且能够发挥良好的镇痛作用。本实验组前期研究发现，硬膜外使用小剂量的右美托咪定可能对术后胃肠运动功能的恢复具有一定的益处。  根据这些研究的结果我们推测，硬膜外使用小剂量的右美托咪定与局麻药，能够发挥良好的  术后镇痛效果，同时与吗啡相比，有利于患者术后胃肠运动功能的恢复。  **部分参考文献**  1 Fukuda H, Tsuchida D, Koda K, Miyazaki M, Pappas TN, Takahashi T. Impaired gastric motor activity after abdominal surgery in rats. Neurogastroenterol Motil 2005; 17: 245-50  2 Holte K, Kehlet H. Postoperative ileus: a preventable event. Br J Surg 2000; 87: 1480-93  3 Bonnet F, Vesinet C. [How can we improve the efficacy of morphine analgesia without increasing adverse effects?]. Cah Anesthesiol 1994; 42: 191-4  4 Thorn SE, Wattwil M, Naslund I. Postoperative epidural morphine, but not epidural bupivacaine, delays gastric emptying on the first day after cholecystectomy. Reg Anesth 1992; 17: 91-4  5 Gillan MG, Pollock D. Acute effects of morphine and opioid peptides on the motility and responses of rat colon to electrical stimulation. Br J Pharmacol 1980; 68: 381-92  6 Porreca F, Filla A, Burks TF. Spinal cord-mediated opiate effects on gastrointestinal transit in mice. Eur J Pharmacol 1982; 86: 135-6  7 Ebert TJ, Hall JE, Barney JA, Uhrich TD, Colinco MD. The effects of increasing plasma concentrations of dexmedetomidine in humans. Anesthesiology 2000; 93: 382-94  8 Esmaoglu A, Yegenoglu F, Akin A, Turk CY. Dexmedetomidine added to levobupivacaine prolongs axillary brachial plexus block. Anesth Analg 2010; 111: 1548-51  9 Rancourt MP, Albert NT, Cote M, Letourneau DR, Bernard PM. Posterior tibial nerve sensory blockade duration prolonged by adding dexmedetomidine to ropivacaine. Anesth Analg 2012; 115: 958-62  10 Salgado PF, Sabbag AT, Silva PC, et al. [Synergistic effect between dexmedetomidine and 0.75% ropivacaine in epidural anesthesia]. Rev Assoc Med Bras 2008; 54: 110-5  11 El-Hennawy AM, Abd-Elwahab AM, Abd-Elmaksoud AM, El-Ozairy HS, Boulis SR. Addition of clonidine or dexmedetomidine to bupivacaine prolongs caudal analgesia in children. Br J Anaesth 2009; 103: 268-74  12 Kanazi GE, Aouad MT, Jabbour-Khoury SI, et al. Effect of low-dose dexmedetomidine or clonidine on the characteristics of bupivacaine spinal block. Acta Anaesthesiol Scand 2006; 50: 222-7  13 Mohamed AA, Fares KM, Mohamed SA. Efficacy of intrathecally administered dexmedetomidine versus dexmedetomidine with fentanyl in patients undergoing major abdominal cancer surgery. Pain Physician 2012; 15: 339-48  14 Zeng XZ, Xu YM, Cui XG, Guo YP, Li WZ. Low-dose epidural dexmedetomidine improves thoracic epidural anaesthesia for nephrectomy. Anaesth Intensive Care 2014; 42: 185-90  15 Aydin C, Bagcivan I, Gursoy S, Altun A, Topcu O, Koyuncu A. Altered spontaneous contractions of the ileum by anesthetic agents in rats exposed to peritonitis. World J Gastroenterol 2009; 15: 1620-4  16 Iirola T, Vilo S, Aantaa R, et al. Dexmedetomidine inhibits gastric emptying and oro-caecal transit in healthy volunteers. Br J Anaesth 2011; 106: 522-7  17 Asai T, Mapleson WW, Power I. Differential effects of clonidine and dexmedetomidine on gastric emptying and gastrointestinal transit in the rat. Br J Anaesth 1997; 78: 301-7 |
| --- |

**五、研究目标**

| **（一）总体目标** 提供良好的术后镇痛效果同时利于术后胃肠运动功能的恢复。 |
| --- |

**六、课题设计**

| **（一）研究内容与方法（此部分为重点阐述内容，包括有关研究方法、实验手段、关键技术、临床研究的研究对象、样本量、入排标准、观察指标和时点等）**    方法：  选择74例ASA I~II级，拟择期行结肠切除术的患者，评估患者符合纳入标准后，与患者签署知情同意书。所有患者均采用全身麻醉联合硬膜外麻醉完成手术。为保证对患者评估的客观准确，所有纳入本研究的患者均由同一组外科医生实施手术。所有患者均行开腹手术，肠道断端使用吻合器吻合。患者围术期接受标准的结肠切除术护理。术后首个24 h可以口服少量清水，第二个24 h可以进食少量流质食物，首次排气后可以开始少量进食固体食物，整个过程由不了解分组情况的同一名外科医师随时评估并指导。  排除标准：神经系统疾病或精神疾病病史，局麻药过敏史，开腹手术史，胃肠功能紊乱病史，肝肾功能不全者，凝血功能异常者，正接受抗凝治疗者。  根据计算机编码将患者随机分为吗啡组（M组）、右美托咪定组（D 组）。两组患者在外科医师开始缝合腹膜时给予不同的硬膜外镇痛药物。吗啡组：硬膜外腔单次给予3 ml吗啡溶液（0.03 mg/kg），随后连接镇痛泵进行48 h的硬膜外术后镇痛，药物配置为：4.5 mg吗啡溶解于总量为150 ml的0.125%左旋布比卡因。右美托咪定组：硬膜外腔单次给予3 ml右美托咪定溶液（0.5 μg/kg），随后连接镇痛泵进行48h的硬膜外术后镇痛，药物配置为：80 μg右美托咪定溶解于总量为150 ml的0.125%左旋布比卡因。  具体实验步骤：  1，患者的疼痛评分采用11分等级疼痛评分体系（VRS），术前向患者详细介绍该评分系统的规则，直至患者完全清楚该评分系统如何运行。  2，所有患者均未给予麻醉前用药。患者入手术室后接受常规的监测，包括心率(HR)、无创血压（Noninvasive blood pressure）、脉搏氧饱和度(SpO_2_)及呼吸频率（Respiratory Rate），静脉给予咪达唑仑2 mg、芬太尼0.05 mg。5 min后，记录上述监测指标数值作为基础值，同时测量患者术前的痛阈（PTh）、疼痛忍受阈（PTTh），具体方法如下：于患者的左前臂放置一刺激电极，产生频率为50-Hz的刺激电流。电流强度从0 mA开始，每一秒钟增加0.1 mA，直至患者感觉到疼痛，记录此时的电流值为PTh；继续增加电流的强度，直至患者感觉疼痛无法忍受，记录此时的电流值为PTTh。10分钟后重复该过程，取两次值的平均值作为患者术前的PTh和PTTh。  3，所有患者均选择T_10-11_硬膜外间隙，经正中入路置入硬膜外导管。应用注射盐水无阻力法确定硬膜外穿刺针是否到达硬膜外腔。置管成功后，硬膜外腔注入 2%的利多卡因+1:200000肾上腺素混合溶液3 ml，以测试硬膜外导管是否误入血管或蛛网膜下腔。经硬膜外导管给予0.33%左旋布比卡因，用针戳法评估感觉神经阻滞范围，当感觉平面达到T_6_时开始实施全身麻醉诱导。麻醉诱导采用静脉注射芬太尼3 μg/kg ，丙泊酚2 mg/kg，维库溴铵0.1 mg/kg。气管插管后连接呼吸机行机械通气，吸入氧浓度为50%，新鲜气流量为2 L/min。全麻维持采用吸入1.3%~2.0%七氟烷，间断给予维库溴铵（0.1 mg/(kg∙h)）维持肌肉松弛。硬膜外腔间断给予0.33%左布比卡因5 ml/h直至手术结束。经右侧锁骨下静脉置入7F中心静脉导管用于围术期补液以及术后静脉营养。当外科医师开始缝合腹膜时，实施上述硬膜外镇痛方案。  记录与评估：  麻醉操作以及所有的记录、评估等工作均由不清楚局麻药配置情况的另一名麻醉医师实施。记录术中及术后6 h内的心率 (HR)、收缩压（SAP）、舒张压（DAP）和平均动脉压（MAP）。采用11分等级疼痛评分体系（VRS）（0=完全不痛，10=能够想到的最痛）评估术后疼痛程度。分别记录患者术后2、4、6、8,、16、24 h 以及 48 h在安静状态以及咳嗽时的疼痛评分。在患者需要时静脉注射氟比洛芬酯100 mg/次，用于控制镇痛不足的术后疼痛。记录两组患者首次应用氟比洛芬酯的时间以及应用氟比洛芬酯的总量。记录患者首次排气以及首次排便发生的时间用于评估术后胃肠运动功能的恢复情况。如果患者首次排气发生在术后6 h以内，则认为排出的气体为残留在直肠内的气体而并不能代表胃肠运动功能的恢复。记录该例患者第二次排气的时间作为胃肠运动功能恢复的标志。记录低血压（MAP低于基础值的30%并持续60 S）、心动过缓(HR低于50次/min）、低氧血症 (SpO_2_ 低于90%)、恶心、呕吐、皮肤瘙痒等不良反应的发生情况。为评估硬膜外镇痛的安全性，分别于术后24、48、72 h 以及7 d时评估患者有无神经系统并发症（神经阻滞区域出现疼痛、麻木或者肌力减退等）。  选择如下技术路线： |
| --- |
| **（二）技术路线**  **分组**  （结肠癌根治术病人74例）  右美组：给予0.5μg/kg右美托咪啶（3ml），然后连接硬膜外输注泵（总量：150ml；右美浓度：0.53μg/ml；左布比浓度：0.125%）  **术后观察指标**  术后2,4,6,8,16，24,48小时的安静状态下和活动状态下的镇痛评分  术后首次出现排气、排便的时间  术后恶心，呕吐，皮肤瘙痒，低血压，心动过缓的发生率  吗啡组：给予0.03mg/kg吗啡（3ml），然后连接硬膜外输注泵（总量：150ml；吗啡浓度：0.003%；左布比浓度：0.125%） |
| **（三）研究难点与对策**    根据随机表严格按照随机顺序进行入组 |

**七、研究伦理**（涉及人类受试者的研究需填写）

| 1. 临床常用药物  2. 知情同意 |
| --- |
